# Develop ment and validation of a prognostic dynamic nomogram for in-hospital mortality in patients with Stanford type B aortic dissection

**DOI:** 10.3389/fcvm.2022.1099055

**Published:** 2023-01-09

**Authors:** Lin Yang, Yasong Wang, Xiaofeng He, Xuanze Liu, Honggang Sui, Xiaozeng Wang, Mengmeng Wang

**Affiliations:** ^1^College of Medicine and Biological Information Engineering, Northeastern University, Shenyang, Liaoning, China; ^2^Department of Cardiology, General Hospital of Northern Theater Command, Shenyang, Liaoning, China

**Keywords:** Stanford type B aortic dissection, in-hospital mortality, nomogram, prediction model, risk factors

## Abstract

**Background:**

This study aimed to identify the risk factors for in-hospital mortality in patients with Stanford type B aortic dissection (TBAD) and develop and validate a prognostic dynamic nomogram for in-hospital mortality in these patients.

**Methods:**

This retrospective study involved patients with TBAD treated from April 2002 to December 2020 at the General Hospital of Northern Theater Command. The patients with TBAD were divided into survival and non-survival groups. The data were analyzed by univariate and multivariate logistic regression analyses. To identify independent risk factors for in-hospital mortality, multivariate logistic regression analysis, least absolute shrinkage, and selection operator regression were used. A prediction model was constructed using a nomogram based on these factors and validated using the original data set. To assess its discriminative ability, the area under the receiver operating characteristic curve (AUC) was calculated, and the calibration ability was tested using a calibration curve and the Hosmer-Lemeshow test. Clinical utility was evaluated using decision curve analysis (DCA) and clinical impact curves (CIC).

**Results:**

Of the 978 included patients, 52 (5.3%) died in hospital. The following variables helped predict in-hospital mortality: pleural effusion, systolic blood pressure ≥160 mmHg, heart rate >100 bpm, anemia, ischemic cerebrovascular disease, abnormal cTnT level, and estimated glomerular filtration rate <60 ml/min. The prediction model demonstrated good discrimination [AUC = 0.894; 95% confidence interval (CI), 0.850–0.938]. The predicted probabilities of in-hospital death corresponded well to the actual prevalence rate [calibration curve: *via* 1,000 bootstrap resamples, a bootstrap-corrected Harrell’s concordance index of 0.905 (95% CI, 0.865–0.945), and the Hosmer–Lemeshow test (χ^2^ = 8.3334, *P* = 0.4016)]. DCA indicated that when the risk threshold was set between 0.04 and 0.88, the predictive model could achieve larger clinical net benefits than “no intervention” or “intervention for all” options. Moreover, CIC showed good predictive ability and clinical utility for the model.

**Conclusion:**

We developed and validated prediction nomograms, including a simple bed nomogram and online dynamic nomogram, that could be used to identify patients with TBAD at higher risk of in-hospital mortality, thereby better enabling clinicians to provide individualized patient management and timely and effective interventions.

## 1. Introduction

Stanford type B aortic dissection (TBAD) is a rare and life-threatening vascular disease that involves detachment of the descending aorta, sometimes extending to the abdomen (1–3). TBAD is a fatal aortic disease with high mortality and morbidity, numerous complications, and a poor prognosis, requiring timely detection and intervention (3–6). In the United States, the incidence of TBAD was estimated to be 2.9–4.3 cases per 100,000 individuals per year (7), whereas in urban adults in China, the incidence is 2.78 cases per 100,000 individuals per year (8). Aortic dissection is considered to be acute when the symptom period up to diagnosis is ≤14 days and chronic when the symptom period is >14 days (9). The mortality rate of patients diagnosed with acute aortic dissection is high before and after admission (10, 11). Thoracic endovascular aortic repair (TEVAR) has the advantages of being minimally invasive, safe, efficient, and simple and has become the main method for the treatment of TBAD (3). Although the treatment technology of TEVAR has made great progress in recent years, studies have revealed that the total in-hospital mortality rate of TBAD is still 4.3–13% (12–14). In particular, some patients died before admission without being diagnosed, and the incidence of in-hospital deaths may have been underestimated (15). Therefore, it is necessary to identify prognostic factors and carry out targeted interventions based on the above reasons. Unfortunately, to date, there are only a few internationally recognized models and tools for predicting in-hospital mortality risk in patients with TBAD.

A nomogram is a statistical model based on the analysis results of the least absolute shrinkage and selection operator (LASSO) or logistic regression model, which graphically transforms complex regression equations to predict disease outcomes. Nomogram models can be integrated with independent risk factors to obtain the numerical probability of a target event and quantify the risk more accurately (16). It is intuitive, easy to understand, easy to read, and flexible for clinical applications.

Consequently, the aim of the present study was to retrospectively analyze the clinical history, laboratory, and imaging data of TBAD patients in our center from 2002 to 2020, screen independent prognostic factors, and establish a nomogram prediction model, including a simple bed nomogram and an online dynamic nomogram, to facilitate clinicians in assessing the risk of in-hospital death from TBAD and making clinical decisions for targeted interventions.

## 2. Materials and methods

### 2.1. Study population

**FIGURE 1:**
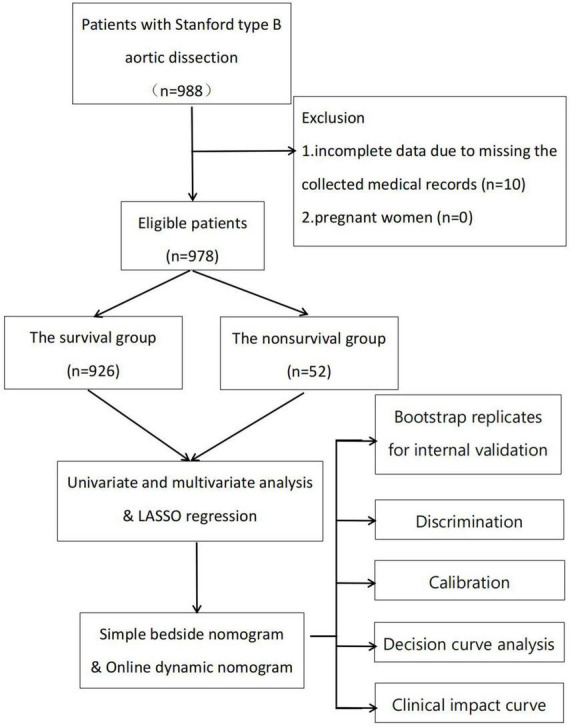
Flow chart of the study. LASSO, least absolute shrinkage and selection operator.

This retrospective observational study was conducted at a single center. Consecutive patients with TBAD at the Department of Cardiology and Cardiovascular Research Institute, General Hospital of Northern Theater Command, between April 2002 and December 2020 were included in the study (Figure 1). The inclusion criteria were: (1) age ≥18 years and (2) diagnosis of TBAD based on the results of aortic computed tomography angiography (CTA) or magnetic resonance angiography (MRA). The following patients were excluded: (1) those with incomplete data due to missing medical records and (2) pregnant women. This study was conducted in accordance with the ethical guidelines of the Declaration of Helsinki. Institutional Review Board approval number: Y (2022) 032; date, March 18, 2022. Because the study was observational and retrospective, the necessity for written informed consent was removed.

### 2.2. Data collection and measures

In the present study, baseline data, including demographics, comorbidities, diagnostic imaging findings, and laboratory values were documented. In our study demographic and patient history information included age, sex, heart rate, arrhythmia, body mass index (BMI), systolic blood pressure (SBP), diastolic blood pressure (DBP), hypotension or shock and history of; cardiac surgery, aortic surgery, chronic obstructive pulmonary disease, liver disease, myocardial infarction, pleural effusion, pericardial effusion, hemorrhagic cerebrovascular disease, ischemic cerebrovascular disease, peripheral vascular disease (PVD), known aneurysm, Marfan syndrome, acute aortic dissection, and diabetes mellitus. The presenting symptoms include new-onset fever, infection, chest pain, abdominal pain, back pain, lumbago, coma, and consciousness disorders. Laboratory tests included hemoglobin, anemia, white blood cells, platelets, C-reactive protein, total cholesterol, triglycerides, high-density lipoprotein, high-density lipoprotein, estimated glomerular filtration rate (eGFR), D-dimer, and abnormal cardiac troponin T (abnormal cTnT). The results of aortic CTA or MRA were also evaluated in patients with TBAD, which included spiral tear, ejection fraction, mean diameter of the descending aorta, maximum diameter of the descending aorta ≥5.5 cm, abdominal vascular involvement, intramural hematoma, and limb ischemia.

The eGFR is recognized as a useful marker for evaluating renal function. eGFR was calculated as follows (17): eGFR (male) = 194 × Scr^–1.094^ × age^–0.287^ ml/min/1.73 m^2^, eGFR (female) = eGFR (male) × 0.739 (Scr: serum creatinine).

Grouping: All included patients were divided into survival and non-survival groups according to whether the patients with TBAD died in-hospital outcomes.

Definition: (1) Infection is defined as a postoperative white blood cell count greater than 9.5 × 10^^9^ (the normal value of laboratory inspection is 3.5–9.5 × 10^^9^) with or without a body temperature exceeding 37.3^°^C. (2) Coma may be defined as a state of prolonged unresponsive unconsciousness. (3) The definition of a “consciousness disorder” usually means that the patient’s response to external stimuli is reduced, accompanied by a state of unresponsiveness, and also accompanied by the loss of sensory and motor functions, but will retain autonomic nervous function. (4) Anemia is defined as hemoglobin levels <12 g/dl in men and <11 g/dl in women. (5) Abnormal cTnT is defined as greater than 0.1 ng/ml (the normal value of a laboratory test is 0–0.1 ng/ml).

### 2.3. Statistical analysis

The baseline characteristics of patients with TBAD were expressed as frequencies and percentages of categorical variables, and as mean ± standard deviation (SD) or median and interquartile range (IQR) (25th or 75th percentiles) for continuous variables. For continuous variables, Kolmogorov-Smirnov test was used to investigate the distribution of the data. In addition, we used the variance inflation factor to assess the collinearity of all variables. The chi-squared test or Fisher’s exact test was used for binary data. Student’s *t*-test was used to compare the significance of the normally distributed data. The Mann-Whitney U-test was used for non-normally distributed data, and median values were compared using the Hodges-Lehmann estimator of location shift with 95% confidence intervals (CI). A univariate logistic regression analysis was performed to evaluate the significance of each variable. Variables with *P* < 0.1 in univariate logistic regression analysis were entered into the multivariate model (forward stepwise logistic regression) to identify independent risk factors. The package “glmnet” was used for the LASSO method to select features as a double check for logistic regression, and factors with non-zero coefficients in the LASSO regression model were selected. Identification of the optimal penalization coefficient λ in the LASSO model with 10-fold cross-validation method and the 1 standard error (1-SE) criterion. A nomogram was constructed to predict the probability of the risk of in-hospital death in patients with TBAD using the package “rms” and “DynNom” in R software (version 4.2.1). The regression coefficients in the multivariate logistic regression analysis were proportionally converted to a point scale, and the total score was converted to the predicted probability.

Nomogram performance was evaluated using discrimination and calibration. The discriminatory ability of the nomogram was evaluated by calculating its area under the receiver operating characteristic (ROC) curve (AUC). The projected and actual probabilities of in-hospital death in TBAD patients were compared by calibration using a visual calibration curve. (18). In addition, it was calibrated using a visual calibration plot with 1,000 bootstrap replicates for internal validation to assess its predictive accuracy, as it is considered more efficient than split data and cross-validation methodologies. Bootstrapping resamples the sample generation process from an underlying population by drawing samples with replacements from the original dataset. The Harrell concordance index (C-index) was used to assess the discriminatory ability of the prediction model. In addition, a web-based dynamic prediction tool for the nomogram was established to facilitate calculations and assist in the clinical decision-making process^1^. Combined with the Hosmer-Lemeshow test, an insignificant Hosmer-Lemeshow test also showed good calibration (*P* > 0.05). We established decision curve analysis (DCA) to assess the clinical usefulness of the generated nomogram by evaluating the net benefits at various threshold probabilities. Based on the DCA curve, a clinical impact curve (CIC) was constructed to further illustrate the predictive ability of the in-hospital mortality risk prediction model. R software (version 4.2.1) and SPSS (version 25.0) were used for statistical and graphical analyses. All tests were two-tailed, and the level of statistical significance was set at *P* < 0.05.

## 3. Results

### 3.1. Baseline characteristics and univariate logistic regression analysis

A total of 978 patients with TBAD were included in this study, of which 52 (5.3%) died in-hospital. The demographic and sociological data between the two groups revealed that BMI in the survival group was (26.06 ± 5.38) kg/m^2^, while (24.89 ± 2.64) kg/m^2^ in the non-survival group (*P* = 0.016). The average age of all patients was 54.28 ± 11.82 years, and approximately one in five were female. The comparison of the demographic and sociological data showed that the mean age in the survival and non-survival groups were 56.96 ± 12.10 and 54.13 ± 11.80, respectively (*P* = 0.093). In addition, the number of patients aged ≥70 years in the non-survival group [10 patients (19.2%)] was significantly higher than that in the survival group [91 patients (9.8%)] (*P* = 0.034). The number of patients with a history of aortic surgery in the non-survival group [three patients (5.77%)] was significantly higher than that in the survival group [eight patients (0.86%)] (*P* = 0.005). In the non-survival group, a significantly higher number of patients [41 patients (78.75%)], had an SBP ≥160 mmHg than in the survival group [398 patients (42.98%)] (*P* < 0.001). Significantly more patients had pleural effusion in the non-survival group than that in the survival group {pleural effusion: non-survival group [32 (61.54%) patients] vs. survival group [160 (17.28%) patients], *P* < 0.001}. In addition, a significant number of patients had pericardial effusion in the non-survival group compared to the survival group {pericardial effusion: non-survival group [9 (17.31%) patients] vs. survival group [45 (4.86%) patients], *P* < 0.001}. A greater number of patients with ischemic cerebrovascular disease was also observed in the non-survival group than in the survival group {ischemic cerebrovascular disease: non-survival group [10 (19.23%) patients] vs. survival group [85 (9.18%) patients], *P* = 0.019}. Moreover, the number of patients with PVD in the non-survival group was significantly higher than that in the survival group {PVD: non-survival group [four (7.69%) patients] vs. survival group [10 (1.08%) patients], *P* = 0.001}. Therefore, age ≥70 years, BMI, history of aortic surgery, SBP ≥160 mmHg, pleural effusion, pericardial effusion, ischemic cerebrovascular disease, and PVD were included in multivariate logistic regression analyses. However, no significant differences were found in other demographic and sociological variables between the two groups (Table 1).

**TABLE 1:** Demographics and patient history of all patients with Stanford type B aortic dissection.

	In-hospital				
	Overall	Survival group	Non-survival group		
	(*n* = 978)	(*n* = 926)	(*n* = 52)	OR (95% CI)	*P*-value
Age (years, $\bar{\text{x}}$ ± s)	54.28 ± 11.82	54.13 ± 11.80	56.96 ± 12.10	1.021 (0.997–1.046)	0.093
Age ≥ 60 years old (%)	330 (33.74)	309 (33.37)	21 (40.38)	1.353 (0.765–2.393)	0.299
Age ≥ 70 years old (%)	101 (10.33)	91 (9.83)	10 (19.23)	2.185 (1.060–4.501)	**0**.**034**
Female (%)	213 (21.78)	196 (21.17)	17 (32.69)	1.809 (0.992–3.298)	0.053
Heart rate (bpm, $\bar{\text{x}}$ ± s)	83.19 ± 15.37	82.66 ± 15.08	92.65 ± 17.40	1.039 (1.022–1.057)	**<0**.**001**
Heart rate > 100 bpm (%)	119 (12.17)	103 (11.12)	16 (30.77)	3.551 (1.904–6.625)	**<0**.**001**
Arrhythmia (%)	41 (4.19)	36 (3.89)	5 (9.62)	2.630 (0.987–7.010)	0.053
BMI (kg/m^2)^	25.99 ± 5.27	26.06 ± 5.38	24.89 ± 2.64	0.903 (0.831–0.981)	**0**.**016**
SBP (mmHg, $\bar{\text{x}}$ ± s)	157.84 ± 26.21	156.97 ± 25.58	173.25 ± 32.19	1.022 (1.012–1.032)	**<0.001**
SBP ≥ 160 mmHg (%)	439 (44.89)	398 (42.98)	41 (78.85)	0.202 (0.103–0.398)	**<0.001**
DBP (mmHg, $\bar{\text{x}}$ ± s)	89.54 ± 17.21	89.67 ± 17.06	87.23 ± 19.60	0.991 (0.975–1.008)	0.318
Hypotension/shock (%)	23 (2.35)	22 (2.38)	1 (1.92)	0.818 (0.108–6.193)	0.846
Smoking history (%)	599 (61.25)	566 (61.12)	33 (63.46)	1.105 (0.619–1.972)	0.736
Acute aortic dissection (%)	773 (79.04)	731 (78.94)	42 (80.77)	1.120 (0.552–2.273)	0.753
Diabetes mellitus (%)	46 (4.70)	43 (4.64)	3 (5.77)	1.282 (0.384–4.282)	0.686
MFS (%)	7 (0.72)	6 (0.65)	1 (1.92)	3.007 (0.355–25.444)	0.312
Known aneurysm (%)	8 (0.82)	7 (0.76)	1 (1.92)	2.574 (0.311–21.322)	0.381
History of cardiac surgery (%)	15 (1.53)	14 (1.51)	1 (1.92)	1.277 (0.165–9.905)	0.815
History of aortic surgery (%)	11 (1.12)	8 (0.86)	3 (5.77)	7.026 (1.807–27.309)	**0**.**005**
History of COPD (%)	49 (5.01)	44 (4.75)	5 (9.62)	2.132 (0.808–5.628)	0.126
History of liver disease (%)	26 (2.66)	25 (2.70)	1 (1.92)	0.720 (0.096–5.422)	0.750
Myocardial infarction (%)	31 (3.17)	28 (3.02)	3 (7.77)	1.959 (0.576–6.669)	0.282
Pleural effusion (%)	192 (19.63)	160 (17.28)	32 (61.54)	7.630 (4.255–13.683)	**<0**.**001**
Pericardial effusion (%)	54 (5.52)	45 (4.86)	9 (17.31)	4.093 (1.879–8.914)	**<0**.**001**
Hemorrhagic cerebrovascular disease (%)	20 (2.04)	19 (2.05)	1 (1.92)	0.954 (0.125–7.268)	0.963
Ischemic cerebrovascular disease (%)	95 (9.71)	85 (9.18)	10 (19.23)	2.379 (1.15–4.918)	**0**.**019**
PVD (%)	14 (1.43)	10 (1.08)	4 (7.69)	7.787 (2.355–25.752)	**0**.**001**

BMI, body mass index; SBP, systolic blood pressure; DBP, diastolic blood pressure; MFS, marfan syndrome; COPD, chronic obstructive pulmonary disease; PVD, peripheral vascular disease. Bold values indicates *P* < 0.05.

The comparison of presenting symptom data between the two groups revealed that coma in the non-survival group [two patients (3.85%)] was higher than that in the survival group [five patients (0.54%)] (*P* = 0.019). Other presenting symptoms data, including new-onset fever, infection, chest pain, abdominal pain, back pain, lumbago, and consciousness disorder, showed no statistical differences (Table 2). Consequently, the multivariate logistic regression analysis included the coma.

**TABLE 2:** Presenting symptoms of all patients with Stanford type B aortic dissection.

	In-hospital				
	Overall	Survival group	Non-survival group		
	(*n* = 978)	(*n* = 926)	(*n* = 52)	OR (95% CI)	*P*-value
**Presenting**					
New onset fever (%)	445 (45.50)	422 (45.57)	23 (44.23)	0.947 (0.540–1.662)	0.850
Infection (%)	69 (7.06)	66 (7.13)	3 (5.77)	0.798 (0.242–2.628)	0.710
Chest pain (%)	691 (70.65)	658 (71.06)	33 (63.46)	0.707 (0.395–1.266)	0.244
Abdominal pain (%)	165 (16.87)	154 (16.73)	11 (21.15)	1.345 (0.676–2.675)	0.398
Back pain (%)	571 (58.38)	545 (58.86)	26 (50.00)	0.699 (0.400–1.223)	0.210
Lumbago (%)	70 (7.16)	68 (7.34)	2 (3.85)	0.505 (0.120–2.119)	0.350
Coma (%)	7 (0.72)	5 (0.54)	2 (3.85)	7.360 (1.393–38.877)	**0.019**
Consciousness disorder (%)	41 (4.19)	37 (4.00)	4 (7.69)	2.002 (0.686–5.847)	0.204

Bold values indicates *P* < 0.05.

Comparison of the diagnostic imaging findings during hospitalization between the two groups revealed that spiral tear, ejection fraction, mean diameter of the descending aorta, maximum diameter of the descending aorta ≥5.5 cm, abdominal vascular involvement, intramural hematoma, and limb ischemia were not significantly different (Table 3).

**TABLE 3:** Diagnostic imaging findings of all patients with Stanford type B aortic dissection.

	In-hospital				
	Overall	Survival group	Non-survival group		
	(*n* = 978)	(*n* = 926)	(*n* = 52)	OR (95% CI)	*P*-value
**Imaging findings**					
Spiral tear (%)	397 (40.59)	381 (41.14)	16 (30.77)	0.636 (0.348–1.162)	0.141
Ejection fraction (%)	59.40 ± 7.77	59.29 ± 7.73	61.35 ± 8.16	1.035 (0.998–1.073)	0.063
Mean diameter of descending aorta (mm, $\bar{\text{x}}$ ± s)	29.67 ± 3.77	29.69 ± 3.72	29.38 ± 4.54	0.978 (0.906–1.056)	0.573
Maximum diameter of descending aorta ≥5.5 cm (%)	25 (2.56)	23 (2.48)	2 (3.85)	1.570 (0.360–6.849)	0.548
Abdominal vascular involvement (%)	395 (40.39)	380 (41.04)	15 (28.85)	0.583 (0.315–1.076)	0.085
Intramural hematoma (%)	16 (1.64)	15 (1.62)	1 (1.92)	1.191 (0.154–9.193)	0.867
Limb ischemia (%)	64 (6.54)	61 (6.59)	3 (5.77)	0.867 (0.263–2.863)	0.815

Bold values indicates *P* < 0.05.

The comparison of laboratory examination data at hospital admission between the two groups showed that the proportion of anemia, abnormal cTnT, and eGFR <60 ml/min in the non-survival group was significantly higher than that in the survival group [anemia: 23 patients in the non-survival group (44.23%) vs. 136 patients in the survival group (14.67%), *P* < 0.001; abnormal cTnT: 14 patients in the non-survival group (26.92%) vs. 48 patients in the survival group (5.18%), *P* < 0.001; eGFR < 60 ml/min: 23 patients in the non-survival group (44.23%) vs. 147 patients in the survival group (15.87%), *P* < 0.001]. At admission, there were no significant differences between the two groups in the results of other laboratory examination data (Table 4). Therefore, anemia, abnormal cTnT levels, and eGFR <60 ml/min were included in the multivariate logistic regression analysis.

**TABLE 4:** Laboratory examinations of all patients with Stanford type B aortic dissection.

	In-hospital				
	Overall	Survival group	Non-survival group		
	(*n* = 978)	(*n* = 926)	(*n* = 52)	OR (95% CI)	*P*-value
**Laboratory examinations**					
Hb (g/L, $\bar{\text{x}}$ ± s)	132.41 ± 18.14	133.13 ± 17.45	119.63 ± 24.61	0.965 (0.952–0.979)	**<0**.**001**
Anemia (%)	159 (16.26)	136 (14.67)	23 (44.23)	4.607 (2.588–8.201)	**<0**.**001**
WBC (10^9^/L, $\bar{\text{x}}$ ± s)	10.37 ± 3.76	10.40 ± 3.78	9.81 ± 3.25	0.955 (0.88–1.036)	0.268
WBC count > 15 × 10^9^/L (%)	99 (10.12)	95 (10.26)	4 (7.69)	0.729 (0.257–2.066)	0.552
PLT (10^9^/L, $\bar{\text{x}}$ ± s)	198.24 ± 79.14	198.98 ± 80.03	185.10 ± 60.31	0.997 (0.993–1.002)	0.219
C-reactive protein (mg/L, $\bar{\text{x}}$ ± s)	43.52 ± 36.18	44.04 ± 36.65	34.27 ± 26.61	0.996 (0.991–1.002)	0.224
TC (mmol/L, $\bar{\text{x}}$ ± s)	4.30 ± 1.66	4.30 ± 1.67	4.34 ± 1.22	1.013 (0.843–1.218)	0.887
TG (mmol/L, $\bar{\text{x}}$ ± s)	1.39 ± 0.93	1.40 ± 0.94	1.19 ± 0.48	0.675 (0.369–1.236)	0.203
HDL (mmol/L, $\bar{\text{x}}$ ± s)	1.27 ± 0.38	1.27 ± 0.40	1.21 ± 0.35	0.860 (0.285–2.598)	0.790
LDL (mmol/L, $\bar{\text{x}}$ ± s)	2.37 ± 0.72	2.36 ± 0.72	2.37 ± 0.80	1.012 (0.613–1.67)	0.964
eGFR < 60 ml/min (%)	170 (17.38)	147 (15.87)	23 (44.23)	0.238 (0.134–0.423)	**<0**.**001**
D-dimer ≥ 5.44 μg/ml (%)	2 (42.45)	22 (2.38)	2 (3.85)	0.637 (0.146–2.777)	0.548
Abnormal cTnT (%)	62 (6.34)	48 (5.18)	14 (26.92)	6.739 (3.420–13.277)	**<0**.**001**

Hb, hemoglobin; WBC, white blood cell; PLT, platelets; TC, total cholesterol; TG, triglycerides; HDL, high density lipoprotein; LDL, low density lipoprotein; eGFR, estimate glomerular filtration rate; cTnT, cardiac troponin T. Bold values indicates *P* < 0.05.

### 3.2. Multivariate logistic regression and LASSO regression analysis

**FIGURE 2:**
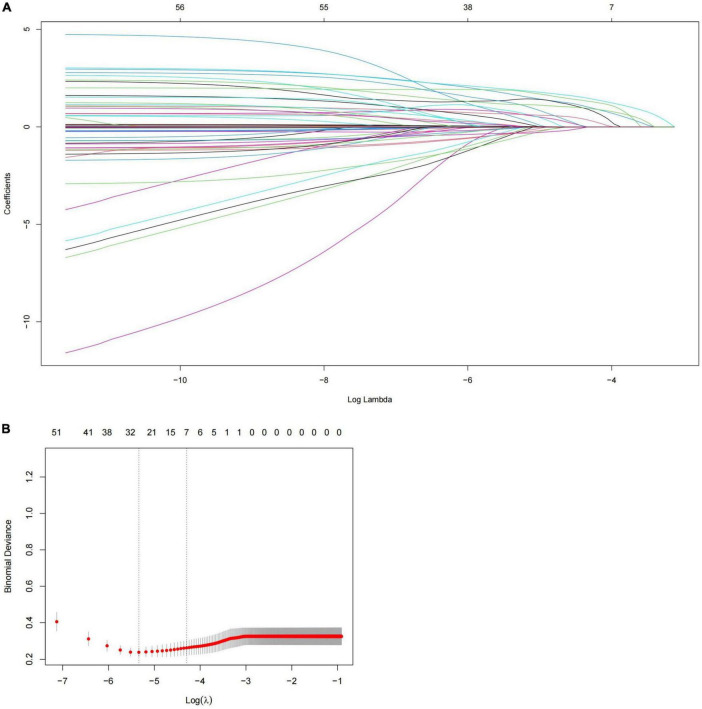
Predictors selection using LASSO regression. **(A)** LASSO coefficient profiles of all the clinical features. **(B)** Identification of the optimal penalization coefficient λ in the LASSO model with 10-fold cross validation and the 1-SE criterion. Results of LASSO regression screening for risk factors for in-hospital mortality. SE, standard error; LASSO, least absolute shrinkage and selection operator.

The analysis’s variables included considerable clinical features and risk factors. The results of the *P* < 0.1 univariate analysis of the variables were used in the multivariate logistic regression analysis. Our study showed that the variables such as age ≥70 years, heart rate >100 bpm, BMI, SBP ≥160 mmHg, history of aortic surgery, pleural effusion, pericardial effusion, ischemic cerebrovascular disease, PVD, coma, anemia, eGFR <60 ml/min, and abnormal cTnT levels, were therefore included in the multivariate logistic regression analysis because they were significantly different in the univariate logistic regression analysis (*P* < 0.05). Seven risk factors were identified using stepwise forward selection to independently predict in-hospital death in patients with TBAD. The result showed that heart rate >100 bpm (OR, 4.444; 95% CI, 2.059–9.594; *P* < 0.001), SBP ≥160 mmHg (OR, 5.678; 95% CI, 2.560–12.597; *P* < 0.001), eGFR <60 ml/min (OR, 2.395; 95% CI, 1.184–4.844; *P* = 0.015), anemia (OR, 3.855; 95% CI, 1.895–7.842; *P* < 0.001), abnormal cTnT (OR, 2.802; 95% CI, 1.182–6.646; *P* = 0.019), ischemic cerebrovascular disease (OR, 3.438; 95% CI, 1.412–8.372; *P* = 0.007), and pleural effusion (OR, 7.858; 95% CI, 3.986–15.493; *P* < 0.001) were independent risk factors for in-hospital death in patients with TBAD (Table 5). To identify the predictors again, we also performed LASSO regression, which showed seven of the same predictors as the logistic regression analysis were selected, thus reinforcing our model (Figure 2).

**TABLE 5:** Multivariate logistic regression analysis of factors possibly associated with in-hospital mortality in tpatients with Stanford type B aortic dissection.

Model variables	OR (95% CI)	Wald	Parameter coefficient	*P*-value
Heart rate > 100 bpm	4.444 (2.059–9.594)	14.436	1.492	**<0**.**001**
SBP ≥ 160 mmHg	5.678 (2.560–12.597)	18.249	1.737	**<0**.**001**
eGFR < 60 ml/min	2.395 (1.184–4.844)	5.909	0.873	**0**.**015**
Anemia	3.855 (1.895–7.842)	13.867	1.349	**<0**.**001**
Abnormal cTnT	2.802 (1.182–6.646)	5.470	1.030	**0**.**019**
Ischemic cerebrovascular disease	3.438 (1.412–8.372)	7.397	1.235	**0**.**007**
Pleural effusion	7.858 (3.986–15.493)	35.437	2.062	**<0**.**001**

SBP, systolic blood pressure; eGFR, estimate glomerular filtration rate; cTnT, cardiac troponin T. Bold values indicates *P* < 0.05.

### 3.3. Nomogram model construction

**FIGURE 3:**
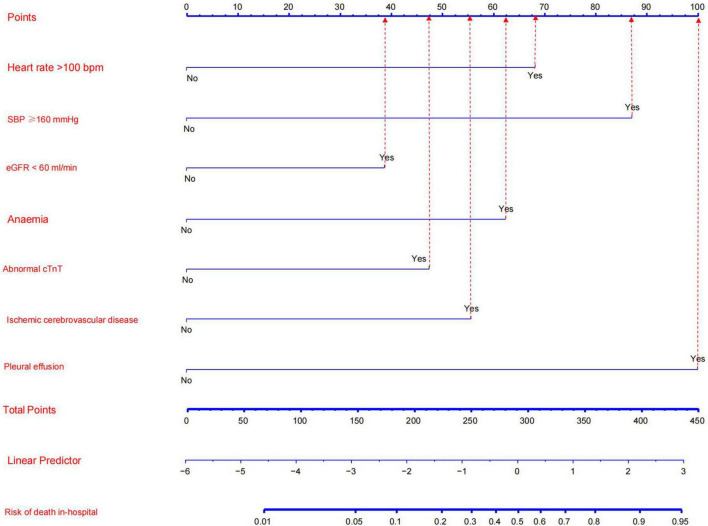
Nomogram model for predicting the risk of in-hospital death in patients with Stanford type B aortic dissection. Every factor in the nomogram got an individual score according to the value of factor, and a total score was obtained by summarizing the scores of the seven factors, which could be used to estimate the probability of the risk of in-hospital death in this type B aortic dissection patient.

**FIGURE 4:**
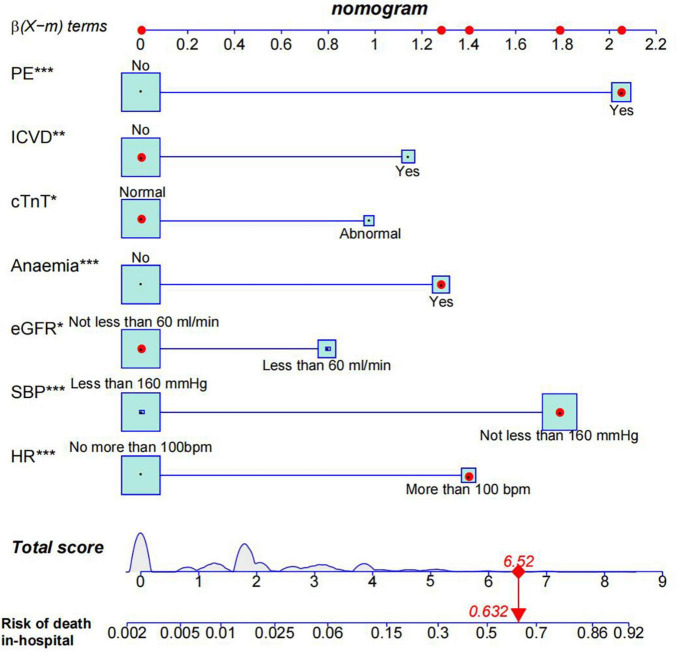
Nomogram model predicts in-hospital death risk in patients with Stanford type B aortic dissection. Given values of the seven variables, the patient can be mapped onto the nomogram. Each red dot represents the value of each variable of the patient. As shown, a probability of 63.2% was the risk of in-hospital death in this type B aortic dissection patient. PE, pleural effusion; ICVD, ischemic cerebrovascular disease; HR, heart rate. *0.01 = *p* < 0.05; **0.001 = *p* < 0.05; ***p < 0.001.

The results of the multivariate logistic regression and LASSO regression analyses were used to build a model that included these independent predictive factors. The model was than displayed as a nomogram, including seven significant independent risk factors: heart rate > 100 bpm, SBP ≥ 160 mmHg, pleural effusion, ischemic cerebrovascular disease, anemia, eGFR < 60 mL/min, and abnormal cTnT levels. To use a nomogram, the subject’s heart rate was first positioned on the axis of correlation. Following that, a straight line was drawn upward to the top point axis to acquire the points based on the heart rate. For each variable, this process was repeated. The total points were than calculated by adding the scores from each covariate. Each predictor’s regression coefficient was scaled to a number of points between 0 and 100, with each point representing the predictor’s relative importance (weight). Points were translated into probabilities using a logit transformation. The probability of in-hospital mortality in a patient was calculated by summing the scores for all variables, and the risk of in-hospital death in patients with TBAD ranged from 0.01 to 0.95 on the nomogram (Figure 3). In addition, a web-based dynamic prediction tool for the nomogram was established to facilitate calculation and assist the clinical decision-making process^1^. For example, for a male patient with heart rate 120 beats/min, blood pressure 170/90 mmHg, eGFR 62 ml/min, hemoglobin 78 g/L, cTnT 0.03 μg/L, pleural effusion, and no ischemic cerebrovascular disease, the total score was approximately 68 + 87 + 0 + 62 + 0 + 0 + 100 = 317, and the predicted risk of in-hospital death for patients with type B dissection was between 0.6 and 0.7 (Figure 3). Simultaneously, the R software (version 4.2.1) was used to draw the dynamic nomogram of the patient, and the results showed that the predicted risk of in-hospital death was 0.632 (Figure 4), indicating that the patient had a high risk of in-hospital death.

### 3.4. Assessment and validation of the nomogram model

**FIGURE 5:**
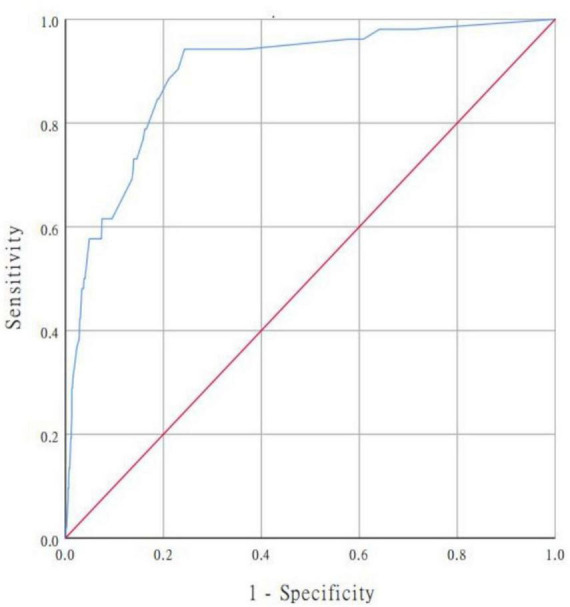
ROC curve for evaluating the model’s discrimination performance. AUC of the ROC curve is 0.894 (95% CI, 0.850–0.938). ROC, receiver operating characteristic; AUC, area under the curve.

**FIGURE 6:**
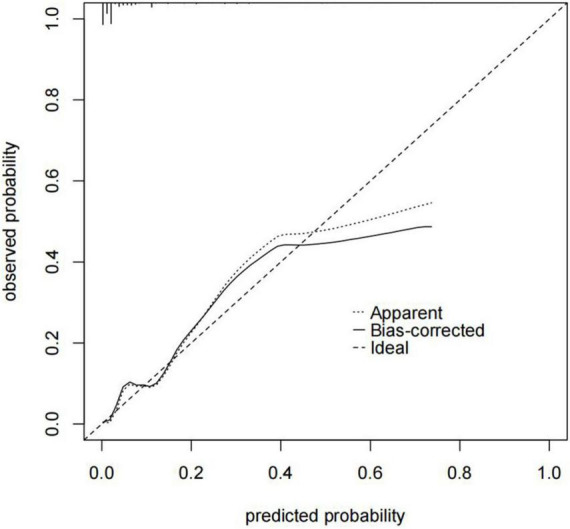
Nomogram calibration curve. The x-axis represents the nomogram-predicted probability, and the y-axis represents the actual probability of the nomogram. The “Ideal” line indicates perfect prediction by an ideal model. The “Apparent” line depicts the model’s performance, and the black solid line is bias-corrected by bootstrapping (B = 1,000 repetitions), indicating observed nomogram performance. C-index of the nomogram calibration curve is 0.905 (95% CI, 0.865–0.945).

In our study, we used a bootstrap method with 1,000 replicates to test the model we constructed. The ROC curve was drawn to assess the predictive capability of the nomogram. The AUC of the nomogram in predicting in-hospital mortality of TBAD was 0.894 (95% CI, 0.850–0.938), suggesting reasonable discrimination (Figure 5). Meanwhile, the calibration curve demonstrated that the in-hospital death probability projected fit well with the actual prevalence rate [calibration curve: *via* 1,000 bootstrap resamples, a bootstrap-corrected C-index of 0.905 (95% CI, 0.865–0.945)] (Figure 6). Futhermore, the non-statistical significance found in the Hosmer–Lemeshow test (χ^2^ = 8.3334, *P* = 0.4016) revealed good calibration.

### 3.5. Clinical use of the nomogram

**FIGURE 7:**
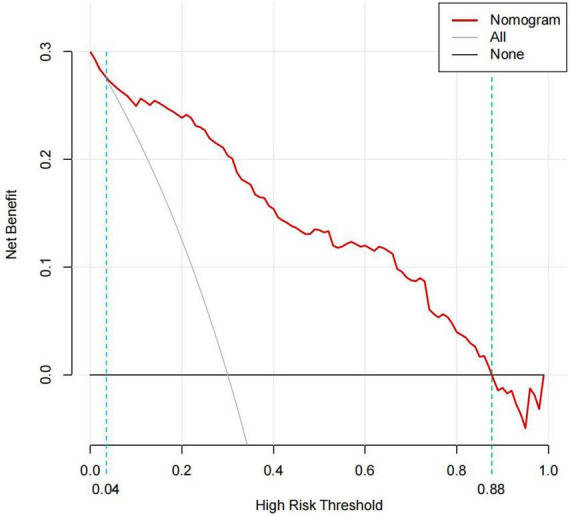
DCA for the predictive nomogram. The y-axis represents the net benefit. The “None” line is the net benefit of intervening no patients. The “All” line is the net benefit of intervening all patients. Solid red line is the net benefit of intervening patients on the basis of the nomogram. The generated curve indicated that at a threshold probability ranging from approximately 4–88%, the nomogram model can be beneficial for making the decision to intervene. DCA, decision curve analysis.

**FIGURE 8:**
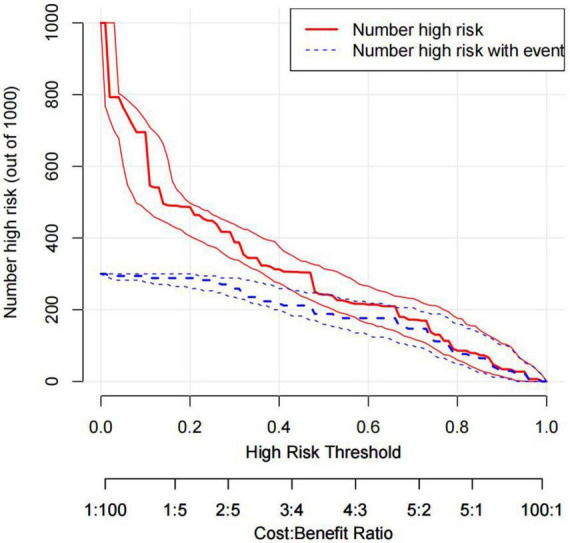
CIC of the nomogram. Two horizontal axes show the correspondence between cost: benefit ratio and risk threshold. Of 1,000 patients, solid red line shows the total number of high-risk patients for each risk threshold. Dotted red line shows how many of those are with positive event. CIC, clinical impact curves.

The DCA is typically used to assess the net clinical benefits of a predictive model. DCA was performed to predict the probability of in-hospital death in patients with TBAD to determine whether this model could provide a significant net benefit. In this study, DCA proved that our predictive model produce more clinical net benefits when the risk threshold was set between 0.04 and 0.88 when compared to the “no intervention” or “intervention for all” options (Figure 7). At the same time, the CIC also demonstrated that the model’s good predictability and clinical applicability (Figure 8).

## 4. Discussion

Several factors have been identified to be related with in-hospital mortality in patients with TBAD (19–25). However, few studies have not only described the independent risk factors for in-hospital mortality in patients with TBAD but have also established a prediction model based on independent risk factors. Therefore, it is essential to devise a trustworthy model to forecast these patients’ prognosis because they are crucial to patient monitoring and care.

Therefore, this study showed great clinical utility, because the nomogram model could predict the risk of in-hospital death in patients with TBAD. Among existing prediction tools, nomograms can easily quantify the risk of in-hospital mortality in patients with TBAD. The nomogram is a valuable clinical tool that integrates easily accessible clinical, imaging, and laboratory data, with good discrimination, calibration, and clinical validity. Seven independent risk factors for in-hospital mortality were identified in the multivariate analysis of 978 patients with TBAD. In the established nomogram, pleural effusion was the most important factor for in-hospital mortality risk in patients with TBAD, followed by, anemia, ischemic cerebrovascular disease, and abnormal cTnT level. An eGFR SBP ≥160 mmHg, heart rate >100 bpm level of <60 ml/min had the least effect on the risk of in-hospital death in patients with TBAD.

The results of the present study indicated that patients with evidence of pleural effusion have higher in-hospital mortality rates. The main causes of pleural effusion in patients with TBAD are rapid tear of the aortic intima, damage to the vessel wall, and blood extravasation, whereas vascular injury, wall inflammation, and edema can lead to reactive exudation, which leads to bloody pleural effusion. A retrospective study showed that pleural effusions appeared on average 4.5 days after the onset of dissection, and thoracentesis showed hemorrhagic effusions in three cases and exudative effusions in three cases. The study also noted that the white blood cell count, serum C-reactive protein level, and body temperature of patients with pleural effusion were higher than those without pleural effusion, suggesting a possible inflammatory reaction (19). Hypoalbuminemia is a recognized cause of pleural effusion and may result from various diseases such as hemodilution, inflammation, and malnutrition (20). The above findings support the conclusions of this study. We believe that the presence of pleural effusion has a greater influence on the respiration and heart rate of patients, which in turn affects their brain, liver, and kidney function.

The present study also revealed that SBP ≥160 mmHg and heart rate >100 bpm were predictors of in-hospital mortality in patients with TBAD. Studies have shown that the target of treatment control for patients with TBAD is a heart rate of 60–80 bpm and an SBP of 100–120 mmHg (21, 22). The reasons for the increased risk of in-hospital death in patients with TBAD can be summarized as follows (23–25): (1) hypertension can promote atherosclerotic changes in the aorta, resulting in increased brittleness of the vascular wall, including intimal thickening and fibrosis, which reduces the elasticity of the vascular wall; (2) hypertension reduces the blood supply of aortic nutrient vessels, resulting in ischemia of the media and the generation of intralaminar shear force; (3) hypertension also promotes the formation of pro-inflammatory cytokines and matrix metalloproteinases, which leads to excessive degradation of the extracellular matrix, easy rupture, and loss of treatment opportunity, leading to an increased risk of in-hospital death; and (4) an increased heart rate is associated with pain, mental tension, increased sympathetic excitability, and hemodynamic instability. Patients with a rapid basal heart rate indicate critical illness, circulatory disturbance, or functional failure, and increase the afterload of the heart, which predicts an increased risk of in-hospital death. The above reasons are also support the results of this study. This study suggests that there is an urgent need to quickly control blood pressure after admission. Effective control of blood pressure and heart rate, reduction of blood pressure fluctuation, and appropriate inhibition of myocardial contraction are the key to treatment. In addition, it is important to improve the early awareness and treatment rates of hypertension to reduce the risk of in-hospital death in patients with aortic dissection.

Ischemic cerebrovascular disease was also a predictor of in-hospital mortality in patients with TBAD. The mechanisms of TBAD complicated by ischemic cerebrovascular disease may be explained as follows (26): (1) retrograde flow with turbulence in the descending aorta; (2) retrograde extension of dissection or false lumen thrombosis; (3) hypotension due to blood loss or shock resulting from malperfusion of other organs; (4) reversible posterior leukoencephalopathy secondary to severe hypertension; and (5) iatrogenic hypotension during the medical management of dissection. Few studies have evaluated ischemic cerebrovascular disease in patients with TBAD, most of which were single-center case reports, and only a small number of patients with ischemic cerebrovascular disease have been evaluated. Patients with ischemic cerebrovascular disease were older, often had hypertension and atherosclerosis, and had symptoms such as syncope, rather than more typical chest or back pain. These patients may also experience hypotension or shock and are more likely to require a longer hospital stay. Similar to the results of our study, in-hospital mortality was higher in patients with ischemic cerebrovascular disease than in those without. Therefore, all patients with ischemic cerebrovascular disease should undergo extensive workup, including, but not limited to, brain CT, MRI, and extremity Doppler ultrasonography.

Anemia was a predictor of in-hospital fatality in TBAD patients in this study. According to WHO criteria, anemia is defined as hemoglobin levels < 13 g/dl in men and <12 g/dl in women (27). However, in China, the definition is distinct because of the region’s unique ethnic and geographical characteristics, with a hemoglobin level threshold of 12 g/dl for men and 11 g/dl for women (28). The following viewpoints may be used to infer explanations for the association between TBAD and hemoglobin levels: first, insufficient tissue oxygenation due to anemia may play a crucial role, thereby leading to aortic dysfunction. In addition, patients with anemia are more likely to experience myocardial ischemia, malnutrition, hemodilution, and renal insufficiency (29). A recent study investigated the relationship between hemoglobin levels and in-hospital mortality in 93 patients with type B dissection and showed that postoperative hemoglobin decline and hemoglobin levels were independent predictors of in-hospital mortality (30). According to our results, anemia was associated with in-hospital death in patients with TBAD, as set forth in previous studies.

Interestingly, we also found that abnormal cTnT level was a predictor of in-hospital mortality risk in TBAD patients. Aortic dissection is a hematoma in the medial aortic layer. It was assumed that cTnT is exclusively expressed in myocardial cells and not in vascular smooth muscle cells; therefore, cTnT levels do not increase in patients with aortic dissection. However, some studies have shown that many patients with aortic dissection have elevated cTnT levels (31, 32), and the mechanism underlying elevated cTnT levels remains unclear. Ventricular overload and stimulation from vasoactive chemicals and inflammatory cytokines may have a synergistic impact, causing myocardial damage and high cTnT levels. A study showed that the concentration of high-sensitivity cTnT in dead patients with Stanford type A acute aortic disease was significantly higher than that in surviving patients. The risk of death significantly increased in the high-sensitivity cTnT (+) group. Therefore, high-sensitivity cTnT levels could be employed as an early biomarker for the risk evaluation of patients with Stanford type A acute aortic disease in the emergency department (33). Another study reported that high-sensitivity cTnT was a strong marker of mortality risk in hospitalized elderly patients (34). In addition, in our study, many patients with TBAD had elevated cTnT levels, which could predict the risk of in-hospital death in patients with TBAD.

The results of the present study suggest that eGFR <60 ml/min is a predictive factor for in-hospital mortality risk in patients with TBAD. Studies have shown that renal dysfunction increases in-hospital mortality and length of hospital stay and decreases late survival (35–38). The possible causes of renal dysfunction can be summarized as follows (39–41): (1) unstable hemodynamic parameters; (2) nephrotoxic drugs and contrast agents are administered to high-risk patients; (3) some patients with TBAD exhibit renal artery involvement; and (4) on admission, the patient had elevated diastolic blood pressure and fasting blood glucose levels. These results support the findings of this study. Based on the results of this study, high-risk patients with confirmed TBAD can be identified if the eGFR is <60 ml/min. Therefore, therapies aimed at improving renal blood flow, such as fenestration or endovascular repair, avoiding the use of nephrotoxic drugs, and optimizing the use of contrast agents, may help reduce in-hospital mortality in patients with TBAD.

It should be admitted that the present study has several limitations First, this analysis was based on data from a single medical center, thus posing a risk of possible patient selection bias. We performed only internal validation by bootstrapping, despite the fact that this model’s internal evaluation revealed excellent calibration and optimal discrimination. External validation, especially from other countries, is lacking given that the clinical manifestations of aortic disease are highly correlated with regional and ethnic differences (42). Second, the prediction model was constructed retrospectively using observational data. Although the number of patients included in this study was not low, it may have had insufficient power to identify other predictors, and the analyses of patient outcomes were based on the results from the initial admission. It is necessary to conduct a larger prospective study to verify this model, which would also update and improve it. Third, because the accuracy of the risk prediction model was derived from a single center, this should be noted when using this model for prediction and making clinical decisions in other centers. Finally, this study included only patients with TBAD. Therefore, our findings may not be applicable to patients with Stanford type A aortic dissection.

## 5. Conclusion

In conclusion, the current study developed and validated prediction nomograms, including a simple bed nomogram and an online dynamic nomogram, that can be used to identify patients with TBAD who may be at higher risk of in-hospital mortality, thereby enabling clinicians to better develop individualized management of patients with TBAD and provide timely and effective interventions.

## Data availability statement

The original contributions presented in this study are included in the article/supplementary material, further inquiries can be directed to the corresponding authors.

## Ethics statement

The studies involving human participants were reviewed and approved by General Hospital of Northern Theater Command. The patients/participants provided their written informed consent to participate in this study.

## Author contributions

LY carried out the studies, participated in collecting data, and drafted the manuscript. YW, XH, XL, and HS participated in the acquisition and analysis or interpretation of data. XW and MW reviewed and edited it. All authors contributed to the interpretation of the data, the completion of figures and tables, and read and approved the final manuscript.

## Footnotes

1
https://amengsci2gogogo.shinyapps.io/dynnomapp/
